# Psychological consequences of COVID-19 pandemic in Italian MS patients: signs of resilience?

**DOI:** 10.1007/s00415-020-10099-9

**Published:** 2020-07-28

**Authors:** Rocco Capuano, Manuela Altieri, Alvino Bisecco, Alessandro d’Ambrosio, Renato Docimo, Daniela Buonanno, Federica Matrone, Federica Giuliano, Gioacchino Tedeschi, Gabriella Santangelo, Antonio Gallo

**Affiliations:** 1 Department of Advanced Medical and Surgical Sciences, University of Campania “Luigi Vanvitelli”, Naples, Italy; 2 Department of Psychology, University of Campania “Luigi Vanvitelli”, Caserta, Italy

**Keywords:** COVID-19, Lockdown, Multiple sclerosis, Anxiety, Depression, Sexual satisfaction

## Abstract

**Background:**

Anxiety, depression and reduction of quality of life (QoL) are common in people with multiple sclerosis (pwMS). Fear of getting sick from COVID-19, government’s lockdown and the imposed social distancing might have had an impact on psychological distress and QoL.

**Objectives:**

The aim of our study was to investigate anxiety, depression and QoL changes in pwMS during SARS-CoV-2 outbreak and lockdown in Italy.

**Methods:**

67 pwMS with a previous (less than 6 months) neuropsychological evaluation before SARS-CoV-2 outbreak (T0) were re-evaluated at the time of the outbreak and lockdown in Italy (T1). They underwent a clinical and neurological evaluation and completed the State-Trait Anxiety Inventory (STAI-Y1), the Beck Depression Inventory second edition (BDI-II), and Multiple Sclerosis Quality of Life-54 (MsQoL-54) at T0 and T1. Benjamini–Hochberg procedure was applied to control the false discovery rate.

**Results:**

BDI-II and STAI-Y1 scores did not change between T0 and T1. At T1, MsQoL-54 scores were higher on the satisfaction with sexual life and the social function subscales, and lower on the limitation due to emotional problems subscale.

**Conclusions:**

This is the first study that evaluated mood and QoL levels before and during the lockdown due to COVID-19 pandemic in pwMS. No worsening of anxiety and depression levels was found. Contrariwise some improvements were noted on QoL, the most reliable regarding the sexual satisfaction and the social function.

## Introduction

Between the end of February and the beginning of March 2020, SARS-CoV-2, the causative agent of COVID-19, quickly spread around the world, endangering the health of people, especially those with older age and/or chronic illnesses [1]. Italy was strongly hit by COVID-19 pandemic, therefore the Italian Government decreed urgent measures promoting social distancing to limit the spread of the virus. In fact, since March 11th, all not indispensable work, social, sporting, retail and recreational activities were suspended or, where possible, converted to the so-called smart-working. Certainly, promoting social distancing is essential to prevent the spreading of SARS-CoV-2 and to ensure public health; on the other hand, changing lifestyle—drastically limiting working and social activities—together with uncertainty and health concerns, might have a significant detrimental effect on mood and mental health.

Multiple Sclerosis (MS) patients, particularly those treated with immunosuppressive drugs, high disability and long-lasting disease might be more susceptible to COVID-19 and its complications, therefore new statement on MS management during SARS-CoV-2 pandemic has been drawn up [2, 3]. Furthermore, mood disorders, particularly anxiety and depression, are common in people with MS (pwMS). Rates of anxiety and depression are higher in pwMS compared with age-matched healthy individuals and patients with other chronic illnesses [4]. Depending on method and study population, prevalence rates for anxiety varies between 20 and 40% [5–8],while the prevalence of depression ranges between 14 and 50% [5, 9–11] with a lifetime prevalence of 50% [11, 12].

Giordano and colleagues reported that, in Italy, 43% of pwMS suffers from anxiety and 34% from depression [13]. Several factors can help explaining the higher prevalence of anxiety and depression in pwMS: (1) the natural reaction to the unpredictable course of the disease, (2) the need to be constantly treated with disease modifying as well as symptomatic drugs, (3) the psychosocial impact of MS in life goals, employment, relationships, and activities of daily living, and (4) disease-related brain structural and functional changes [4, 14–16].

Social and working restrictions imposed by the lockdown as well as the fear of getting severely sick might have influenced anxiety and depression levels of pwMS. The aim of our study was, therefore, to investigate—in a group of pwMS in which a pre-pandemic neuropsychological and behavioral evaluation was available—changes in lifestyle, levels of anxiety, depression and quality of life (QoL) during the Italian lockdown due to SARS-CoV-2 pandemic.

## Methods

Seventy-five relapsing–remitting (RR) MS patients that had a pre-lockdown (T0) neurological, neuropsychological and behavioral evaluation between September and December 2019 as part of the ongoing clinical and/or research activities at the MS Center of the I Division of Neurology of the University of Campania “Luigi Vanvitelli” were selected. Patients were reached at the phone during the lockdown between April 16th and April the 23rd, 2020, when they were invited to participate to a second psychological and behavioral assessment (T1). To be included in the study, patients had to have a diagnosis of RRMS according to last revised McDonald criteria [17]; they had to be relapse- and steroid-free within the month prior to baseline assessment (T0) and during the time-window between baseline and follow-up assessment (T0–T1). The study was approved by the local Ethical Committee. All participants were informed on the objectives of the study and provided informed consent by means of an online form.

### Clinical assessment

At T0, the following data were recorded from each participating subject: the Expanded Disability Status Scale [18] score, the disease duration, and the disease modifying therapy (DMT). DMTs were classified as low/moderate-efficacy DMTs (dimethyl fumarate, glatiramer acetate, interferons, teriflunomide) and moderate/high-efficacy DMTs (alemtuzumab, fingolimod, natalizumab, ocrelizumab).

At T1, we conducted structured telephone calls to enrol patients and, after receiving informed consent, a clinical and neurological assessment evaluating for COVID-19 symptoms. Occurence of relapses and/or DMTs changes, was performed via telephone call by a neurologist with expertise in MS.

### Neuropsychological and behavioral assessment

The following neuropsychological tests and behavioral scales were administered at T0: (1) the Symbol Digit Modalities Test (SDMT) a neuropsychological test that evaluates processing speed efficiency and represents a valuable surrogate marker of global cognitive status [19, 20]. For the purposes of the study, raw scores of SDMT were converted into corrected scores according to the formula provided by Italian normative data [19] and then they were transformed into z-scores. pwMS were considered cognitively impaired if their SDMT z-scores were ≤ 1.5 standard deviations; (2) the State Anxiety section of the Italian version of the State-Trait Anxiety Inventory (STAI-Y-1) [21] to evaluate state-anxiety levels; STAI-Y-1 scores range from 0 to 80 and higher scores are indicative of higher levels of state-anxiety; (3) the Italian version of the Beck Depression Inventory—II Edition (BDI-II) [22] to investigate patients’ depressive symptomatology; the scores range from 0 to 63 and scores higher than 18 are indicative of depressive symptomatology; (4) the Italian version of the Multiple Sclerosis Quality of Life-54 (MSQoL-54) [23] to estimate the QoL. MSQoL-54 provides a number of indices to evaluate different aspects of QoL: physical health, limitations due to physical and emotional problems, pain, emotional well-being, energy, health perceptions, social, cognitive and sexual functions, health distress, change in health, satisfaction with sexual function, overall quality of life. Moreover, composite scores related to physical (PHCs) and mental (MHCs) well-being are provided. The scores of each subscale range from 0 to 100 (lower and higher levels of QoL, respectively).

At T1, a trained neuropsychologist, who tested patients at T0, proceeded by sending an email with a link to an online form. The structured self-assessment consisted of: (1) a survey aimed to investigate lifestyle changes and feelings during the Italian lockdown; out of 20 items listed in the survey, the most relevant explored/regarded: fear of getting COVID-19 having MS and/or being treated with DMTs; change in adherence to DMTs; repercussions on working habits; changes of social habits; self-perception of changes; (2) the Italian version of STAI-Y-1; (3) the Italian version of BDI-II; (4) the Italian male and females versions of MSQoL-54.

### Statistical analyses

All analyses were performed using SPSS (SPSS Statistics version 25.0).

Absolute numbers and percentage were used to describe categorical variables, means and standard deviations (SD) or medians and interquartile ranges (P25-P75) were used for continuous variables.

STAI-Y-1, BDI-II and MSQoL-54 scores at T0 and T1 were compared using the paired *t* test (*t* test for dependent samples) or chi-square where appropriate. A subgroup analysis was performed to evaluate possible differences on depression, state-anxiety and QoL, at T0 and T1, in males and females. Correlations between significant *t* test variables and BDI-II, STAI-Y1, MSQoL-54 subscales, including PHCs and MHCs at T0 and T1 were evaluated by Pearson correlation coefficient.

Three sensitivity analyses were performed by excluding: (1) baseline depressed patient; (2) baseline anxious patients; (3) baseline cognitive-impaired patients. A *p* value of 0.05 was the cut-off for significance. Benjamini–Hochberg procedure was applied to control the false discovery rate [24].

## Results

Sixty-seven out of 75 patients with the T0 evaluation agreed to participate and were enrolled in the study. Two patients declined to participate, while six were not reachable at the time of the survey. Patients’ socio-demographic and clinical characteristics registered at T0 are summarized in Table 1. No participants changed their DMT between the two time-points. Six out of 67 patients (9%) reported symptoms possibly due to SARS-CoV-2 infection, but only 1 (1.5%) underwent a double negative nasopharyngeal swab for diagnosis of COVID-19; any patient nor their family members or cohabitants were diagnosed with COVID-19. Thirty-eight (56.7%) patients reported to feel at higher risk of getting COVID-19 due to MS, while 30 (44.8%) patients reported that the cause of being more at risk of getting COVID-19 was related to being on a DMT. However, the vast majority of the patients (*N* = 66; 98.5%) did not report any change in adherence to DMT schedule; only one patient reduced the adherence but did not stop DMT assumption. Thirty-four (50.7%) patients reported that they contacted a physician to get more information about COVID-19 (6% contacted their family doctor; 34.3% contacted doctors of their MS Center; 10.4% contacted both).

**Table 1:** Socio-demographic and clinical characteristics of Multiple Sclerosis sample before COVID-19 lockdown (N = 67)

Mean Age (years, SD)	37.5 (11.1)
Sex (M/F)	30/37
Mean years of education (SD)	13.7 (3.5)
Mean disease duration (months, SD)	91.5 (96.9)
Median EDSS (P25–P75)	2.0 (1.0–2.0)
Mean SDMT (SD)	49.9 (13.4)
DMTs, *n* (%)	
None	8 (11.9)
Low/moderate efficacy	39 (58.2)
Moderate/high efficacy	20 (29.9)

*SD* standard deviation, *EDSS* Expanded Disability Status Scores, *P25* 25th percentile, *P75* 75th percentile, *SDMT* Symbol Digit Modalities Test, *DMTs* disease modifying therapies

Fifty-seven (85.1%) (*N* = 57) patients reported significant changes in social and lifestyle habits, while 39 (58.3%) reported more difficulties in daily life. Twenty out of 67 patients (29.8%) were unemployed at the time of SARS-CoV-2 outbreak. Only 7 patients (10.5%) continued to work in the same modality as before the lockdown, while 16 (23.9%) worked from home (smart-working) and 24 (35.8%) stopped working at all.

As regards the mood and behavioral status of the patients, 8 patients (11.9%) were depressed and 8 (11.9%) were anxious at T0; at T1, the same prevalence of depression was found, while 11 patients (16.4%) reported state-anxiety. However, no significant increase in anxiety prevalence was observed during the lockdown (16.4%, χ^2^ = 0.488, *p* = 0.485). No differences were found on BDI-II and STAI-Y1 scores between T0 and T1 (Table 2).

**Table 2:** Behavioral and quality of life assessment pre- and during the COVID-19 lockdown in Italy

	T0	T1	*T*	*p*
BDI-II—mean (SD)	9.94 (7.9)	8.6 (9.3)	1.589	.117
STAI-Y1—mean (SD)	42.1 (11.4)	43.5 (12)	– 1.005	.319
MSQoL physical health—mean (SD)	78.5 (26.9)	82.6 (24.4)	– 1.716	.091
MSQoL limitations due to physical problems—mean (SD)	77.4 (35.2)	77.3 (35.2)	.015	.988
MSQoL limitations due to emotional problems—mean (SD)	81.5 (32.7)	67.7 (40.7)	3.102	**.003**
MSQoL pain—mean (SD)	75.4 (25.3)	82 (25)	– 1.876	.065
MSQoL emotional well-being—mean (SD)	64.2 (18.4)	64.3 (21.4)	– .059	.954
MSQoL energy—mean (SD)	51.1 (18.2)	48.1 (17.9)	1.518	.134
MSQoL health perceptions—mean (SD)	54.4 (20.9)	55.9 (21.4)	– .705	.483
MSQoL social function—mean (SD)	77.7 (17.4)	84.2 (20.4)	– 3.133	**.003**
MSQoL cognitive function—mean (SD)	72.7 (18.2)	74.3 (23.6)	– .742	.461
MSQoL health distress—mean (SD)	72.9 (22.4)	74.2 (23.9)	– .621	.537
MSQoL sexual function—mean (SD)	83.3 (26)	87.2 (21.8)	– 1.066	.290
MSQoL change in health—mean (SD)	44.9 (22.3)	53.5 (21.8)	– 2.295	**.025**
MSQoL satisfaction with sexual function—mean (SD)	70.1 (25.7)	89.3 (21.1)	– 5.791	** < .001**
MSQoL overall quality of life—mean (SD)	64.2 (21.3)	68.4 (16.3)	– 1.702	.094
MSQoL physical health composite score—mean (SD)	70.2 (17.8)	72.9 (19.8)	– 1.487	.142
MSQoL mental health composite score—mean (SD)	71.1 (17.7)	68.7 (21.8)	1.278	.206

Values at T0 and T1 are expressed as mean (standard deviation). *T0* before the lockdown, *T1* during the lockdown, *t* paired sample *t* test, *p* probability value, *BDI-II* Beck Depression Inventory, second edition, *STAI-State* State Anxiety section of the State-Trait Anxiety Inventory (STAI-Y-1), *MSQoL-54* Multiple Sclerosis Quality of Life-54. Significant values (*p* < .05) are reported in bold. Significant values after Benjamini–Hochberg procedure are underlined

After correction for multiple comparisons, at T1 the scores of the following subscales of the MsQoL-54 showed significant changes: higher scores on the satisfaction with sexual function subscale (*p* < 0.001), lower scores on the limitations due to emotional problems subscale (*p* = 0.003) and higher scores on the subscales addressing social function (*p* = 0.003).

At T1, a trend was found on changes in perceived health status (*p* = 0.025), but it did not survive the correction for multiple comparison. Patients were asked to explain reasons of improvement and 58.7% attributed the amelioration to increased partner support, 30.4% to reduced work-related stress and 10.9% was not able to specify the reason. Moreover, the satisfaction with sexual function subscale was significantly and negatively correlated with BDI-II and STAI-Y1 scores and positively with both PHCs and MHCs, at T0 (BDI-II: *r* =  − 0.397, *p* = 0.001; STAI-Y1: *r* =  − 0.261, *p* = 0.041; PHCs: *r* = 0.528, *p* < 0.001; MHCs: *r* = 0.482, *p* ≤ 0.001) and T1 (BDI-II: *r* =  − 0.472, *p* < 0.001; STAI-Y1: *r* =  − 0.437, *p* < 0.001; PHCs: *r* = 0.543, *p* < 0.001; MHCs: *r* = 0.579, *p* ≤ 0.001).

Moving on sex differences (Table 3), we found that women scored higher than men on BDI-II (*t* =  − 2.833, *p* = 0.006) and STAI-Y1 (− 3.121, *p* = 0.003) at T0, but not at T1 (BDI-II: *t* = − 0.869, *p* = 0.388; STAI-Y1: *t* = − 1.176, *p* = 0.244). The intragroup analysis (Table 3) to evaluate possible differences between T0 and T1 revealed some trends that lost significance after Benjamini–Hochberg correction: higher STAI-Y1 scores in males at T1 (*t* = − 2.105; *p* = 0.044) and lower BDI-II scores in females at T1 (*t* = 2.162; *p* = 0.037). Moreover, trends between T0 and T1 were found on the following MSQoL-54 subscales (Table 3): pain subscale (*t* = − 3.191, *p* = 0.004) in males; social function subscale and satisfaction with sexual function in both males (*t* = − 2.465, *p* = 0.02; *t* = − 2.491, *p* = 0.019, respectively) and females (*t* = − 2.062, *p* = 0.047; *t* = − 6.068, *p* < 0.001); limitations due to emotional problems subscale and overall quality of life (*t* = 2.443, *p* = 0.020; *t* = − 3.163, *p* = 0.003, respectively) in females. After applying the Benjamini–Hochberg procedure, only the differences between T0 and T1 on the satisfaction with sexual function and overall QoL subscales of MSQoL-54 in females remained significant. Lastly, three sensitivity analysis were performed by excluding, respectively, anxious, depressed and cognitively impaired patients at T0 and no differences were found with respect to the whole sample.

**Table 3:** Behavioral and quality of life (QoL) assessment pre- and during the COVID-19 lockdown in Italy in females and males pwMS

	FEMALES				MALES			
	T0	T1	*t*	*p*	T0	T1	*t*	*p*
BDI-II—mean (SD)	12.3 (9)	9.5 (10.9)	2.162	**.037**	7.03 (5.2)	7.5 (7.40)	− .510	.614
STAI-Y1—mean (SD)	45.81 (10.8)	45 (12.1)	.396	.694	37.5 (10.7)	41.6 (11.76)	− 2.105	**.044**
MSQoL-54 physical health—mean (SD)	78.3 (26.9)	80.7 (28.0)	− .727	.472	78.7 (27.5)	85.0 (19.10)	− 1.803	.083
MSQoL-54 limitations due to physical problems—mean (SD)	80 (34.1)	77.1 (35.5)	.399	.693	74.1 (36.9)	77.7 (35.57)	− .518	.608
MSQoL-54 limitations due to emotional problems—mean (SD)	81.9 (31.8)	67.6 (41.8)	2.443	**.020**	80.9 (34.5)	67.9 (40.04)	1.890	.070
MSQoL-54 pain—mean (SD)	76.2 (25.9)	77.1 (29.4)	− .171	.865	74.3 (24.8)	88.4 (16.11)	− 3.191	**.004**
MSQoL-54 emotional well-being—mean (SD)	59.6 (17.7)	60.1(22.8)	− .290	.835	70.1 (17.8)	69.7 (18.55)	.121	.905
MSQoL-54 energy—mean (SD)	48.1 (17.7)	44.6 (17.4)	1.469	.151	55 (18.3)	52.7 (17.69)	.688	.497
MSQoL-54 health perceptions—mean (SD)	52.6 (19.8)	53.7(22.4)	− .363	.719	56.8 (22.3)	58.6 (19.99)	− .757	.456
MSQoL-54 social function—mean (SD)	76.6 (17.6)	82.9 (23.6)	− 2.062	**.047**	79.2 (17.3)	86.0 (15.56)	− 2.465	**.020**
MSQoL-54 cognitive function—mean (SD)	69.7 (19)	73.9 (24.1)	− 1.574	.124	76.6 (16.7)	74.8 (23.27)	.532	.599
MSQoL health distress—mean (SD)	69.7 (23.7)	70.3 (26.4)	− .226	.823	76.9 (20.5)	79.3 (19.61)	.615	.544
MSQoL sexual function—mean (SD)	80 (29)	87.9 (22.9)	− 1.554	.129	87.5 (21.6)	86.3 (20.69)	.239	.813
MSQoL change in health—mean (SD)	46.5 (22.5)	54.9 (20.5)	− 1.784	.083	42.9 (22.4)	51.8 (23.50)	− 1.441	.161
MSQoL satisfaction with sexual function—mean (SD)	68.2(23.7)	92.4 (15.9)	− 6.068	** < .001**	72.3 (28.3)	85.7 (25.84)	− 2.491	.091
MSQoL overall quality of life—mean (SD)	59.5 (20)	69.2 (15.5)	− 3.163	**.003**	70.2 (21.7)	67.4 (17.61)	.716	.480
MSQoL physical health composite score—mean (SD)	68.8 (17.4)	70.7 (21.5)	− .742	.463	72 (18.5)	75.7 (17.39)	− 1.430	.164
MSQoL mental health composite score—mean (SD)	68.5 (15.9)	67 (22.8)	.680	.501	74.4(19.5)	70.9 (20.65)	1.097	.283

Values at T0 and T1 are expressed as mean (standard deviation). *T0* before the lockdown, *T1* during the lockdown, *t* paired sample *t* test, *p* probability value, *BDI-II* Beck Depression Inventory, second edition, *STAI-Y1* State Anxiety section of the State-Trait Anxiety Inventory, *MSQoL-54* Multiple Sclerosis Quality of Life-54. Significant values (*p* < 0.05) are reported in bold. Significant values after Benjamini–Hochberg procedure are underlined

## Discussion

In the present study, we investigated lifestyle changes together with levels of anxiety, depression and QoL in pwMS during the Italian lockdown due to SARS-CoV-2 pandemic. Notably, we designed the study to enrol only pwMS for whom a recent neuropsychological and behavioural assessment was already available before the COVID-19 lockdown. By designing the study in this way, we were able to have a baseline/reference time-point against which to compare the scores measured during the lockdown. To the best of our knowledge, only anxiety was investigated in a small cross-sectional study conducted on Iranian pwMS during SARS-CoV-2 outbreak [25].

As expected, several patients significantly changed their social and lifestyle habits with more difficulties encountered in daily life due to the country lockdown. More than a half of the patients had to change their work habits by stopping working or starting smart-working, while only the 10.5% of subjects continued working in the same modalities as before the lockdown.

Despite adaptation to home restrictions, no differences in levels of anxiety or depression were found between T1 and T0. These results are in apparent contrast with a recent Italian survey conducted on the general population (age range 18–90 years), which reported a high psychological distress during the lockdown [26]. This difference, indeed, might be explained by one or more of the following: (1) our sample was mostly constituted by young adults with MS; even if all people are at risk of psychological harm when kept in “isolation”, children, adolescents and older adults are the most vulnerable while young adults are the most resilient [27]; (2) since the studied population was from Campania, an Italian region that was affected by the pandemic much less than other northern regions [28], it is possible that levels of fear of getting COVID-19 were lower; (3) pwMS might be more accustomed to live with higher levels of anxiety and depression compared to healthy people, showing a higher resilience to external events concerning all.

As already mentioned, the only preliminary report that investigated anxiety during COVID-19 pandemic was conducted on 33 Iranian pwMS and found high levels of anxiety [25], although the study design did not establish a pre-post comparison, therefore it was not possible to verify if levels of anxiety were already high before the lockdown. On these bases, we should have expected a significant negative impact of the pandemic in our cohort. Despite this, probably due to different study design with a T0/T1 evaluation and a larger cohort studied, we provided evidence that SARS-CoV-2 outbreak did not have a negative impact on anxiety, depression and even improved some aspects of QoL.

Moreover, we have even been able to demonstrate that, during the lockdown, pwMS reported a higher sexual satisfaction, both in the whole and in the females’ group. Since we did not find differences on practical/physiological function between T0 and T1 (assessed by means of the sexual function subscale of MSQoL-54), our results strongly support the psychological component in determining the observed improvement. Sexual satisfaction is strongly related to QoL and may have a positive impact on anxiety and depression [29, 30]. This might also help explaining the observation of a trend toward an improvement of depressive symptoms during the lockdown in female patients. Moreover, men showed a trend of higher state-anxiety levels at T1; this may be due to either a non-significant improvement in sexual satisfaction subscale or to higher females levels of anxiety at T0 [21], limiting them to further worse their anxiety within a short time frame.

In addition, we found that our cohort reported a higher social functioning. According to our survey, being at home and being more able to spend time and interact with their partners (58 out of 67 of our patients lived with a partner) and family members may have led to a higher perceived social support, having a positive impact on sexual satisfaction and mood, in line with previous studies [31–34]. Furthermore, several pwMS experience frustration, anger and other negative emotions because, compared to their healthy peers, they have more limitations in engaging in outdoor activities such as shopping, driving, participating at social and sporting activities [35]. Consequently, we can speculate that, even if they perceived themselves as more limited in daily activities, the lockdown might have been perceived by pwMS as a sort of levelling, because social restrictions caused by COVID-19 lockdown interested all people, regardless of their health status. Moreover, feelings of uncertainty about the future, fear about their own health or anxiety/frustration/anger during pandemic may be more frequent and socially accepted than before [36].

Sensitivity analysis excluding anxious, depressed or cognitive impaired patients at baseline did not change the results and this is a strength of our study.

On the other hand, this study is not without limitations. First, participants are all from Southern Italy. Even if the government decree that enacted the lockdown applied to the whole country, the spreading of COVID-19, as already said, was much slower in the Southern regions than in Northern ones; this might have an impact on the generalizability of our results. Moreover, we found lower percentages of depressed pwMS with respect to other studies [5, 9–11, 13], this result might be due to: (1) the RR phenotype and to the low levels of disability of our patients (median EDSS = 2.0), in fact BDI-II scores is known to correlate with disability and MS disease course [37]; (2) the cut-off score of 18.5, which, although validated in the Italian MS population [22], is higher than other international BDI-II cut-offs [37]. Therefore, our results must be taken with caution in patients with higher levels of disability and/or depressive symptomatology. Another limitation is the absence of a comparator group of healthy subjects. Finally, even if behavioural assessments at both time points were completed with no assistance, modalities of administration differed slightly. At T0, patients completed questionnaires at our MS Center, whereas at T1, they completed questionnaires online at their own homes during the lockdown due to the Hospital rules of that period, which did not permit patients the access at our MS Centre for non-urgent conditions. Different assessment conditions may have affected final assessment result. Finally, sexual satisfaction was evaluated just based on a single/specific item of the MSQoL-54. This preliminary finding could be further explored in future studies.

In conclusion, we provided evidence that despite the tight Italian lockdown due to the COVID-19 pandemic and the fear of getting sick, we did not observe a relevant negative impact on anxiety, depression and QoL of our sample of pwMS. Contrariwise, we were even able to detect some positive effects on specific aspects of QoL that might be also interpreted as signs of resilience.

## Data Availability

The data that support the findings of this study are available from the corresponding author, prof. Antonio Gallo, upon reasonable request.
